# Changes in the Impacts of Topographic Factors, Soil Texture, and Cropping Systems on Topsoil Chemical Properties in the Mountainous Areas of the Subtropical Monsoon Region from 2007 to 2017: A Case Study in Hefeng, China

**DOI:** 10.3390/ijerph18020832

**Published:** 2021-01-19

**Authors:** Qing Li, Fenlan Gu, Yong Zhou, Tao Xu, Li Wang, Qian Zuo, Liang Xiao, Jingyi Liu, Yang Tian

**Affiliations:** The College of Urban and Environmental Sciences, Central China Normal University, Wuhan 430079, China; ennstar@mails.ccnu.edu.cn (Q.L.); gfl1994@yeah.net (F.G.); xutao@mails.ccnu.edu.cn (T.X.); wli_gis@mails.ccnu.edu.cn (L.W.); zuoqccnu@mails.ccnu.edu.cn (Q.Z.); xiaoliang@mails.ccnu.edu.cn (L.X.); liujy21@mails.ccnu.edu.cn (J.L.); tianyang@mails.ccnu.edu.cn (Y.T.)

**Keywords:** spatial variability, soil properties, semivariogram, geostatistical analysis

## Abstract

Understanding the spatial pattern of soil chemical properties (SCPs) together with topological factors and soil management practices is essential for land management. This study examines the spatial changes in soil chemical properties and their impact on China’s subtropical mountainous areas. In 2007 and 2017, 290 and 200 soil samples, respectively, were collected in Hefeng County, a mountainous county in central China. We used descriptive statistics and geostatistical methods, including ANOVA, semivariance, Moran’s I, and fractal dimensions, to analyze the characteristics and spatial autocorrelation changes in soil organic matter (OM), available phosphorus (AP), available potassium (AK), and pH value from 2007 to 2017. We explored the relationship between each SCP and the relationship between SCPs with topographic parameters, soil texture, and cropping systems. The results show that the mean value of soil OM, AP, AK, and pH in Hefeng increased from 2007 to 2017. The spatial variation and spatial dependency of each SCP in 2007, excluding AP and AK in 2007, were higher than in 2017. The soil in areas with high topographic relief, profile curvature, and planform curvature had less AP, AK, and pH. Soil at higher elevation had lower OM (r = −0.197, *p* < 0.01; r = −0.334, *p* < 0.01) and AP (r = −0.043, *p* < 0.05; r = −0.121, *p* < 0.05) and higher AK (r = −0.305, *p* < 0.01; r = 0.408, *p* < 0.01) in 2007 and 2017. Soil OM and AK in 2007 were significantly (*p* < 0.05) correlated with soil texture (*p* < 0.05). In contrast, oil AP and soil pH in 2007 and all SCPs in 2017 were poorly correlated with soil texture. The cropping systems played an important role in affecting all SCPs in 2007 (*p* < 0.01), while they only significantly affected AK in 2017 (*p* < 0.05). Our findings demonstrate that both topological factors, that is, the changes in cropping management and the changes in acid rain, impact soil chemical properties. The local government should place more focus on reducing soil acid amounts, soil AP content, and soil erosion by improving water conservancy facilities.

## 1. Introduction

Soil is the foundation of life and biodiversity [1,2]. Climate change and human intervention speed up soil property change [3]. Soil degradation is a global issue that leads to crop reduction. Understanding soil properties and properly managing soil are critical for avoiding soil degradation [4,5] and achieving the United Nations’ sustainable development goals [6].

In the past, soil degradation in China was serious due to intensive and unsustainable agricultural activities. Soil problems such as soil acidity in South China [7] and soil cadmium in central China [8] threaten national food security. With a population of 1.4 billion people and a rising demand for improved food quality and food structure, the Chinese government is focusing much attention on food security and soil health [9]. Previous research explored the spatial distribution of soil properties and soil fertility in different areas to help local agricultural departments to scientifically adjust soil management [10,11,12]. Soil properties are not only related to soil parent materials but also to climatic, topographic, hydrological [13], and ecological elements [2], as well as human activities [10] and other factors [9]. There is no universal soil property variation pattern. The characteristic of soil property variation in one place is different to that in other areas. For example, Tian and Niu (2015) showed that the widespread use of inorganic N fertilizers is one of the reasons for global soil acidification [14], while Shuai Chen et al.’s research (2020) showed that soil pH value in Jianli county has risen during the period of 2007–2017 [11].

Existing research in China on spatial distribution and the spatial–temporal condition of soil chemical properties is either outdated or does not include the study of soils in subtropical mountains. For example, Bo et al. (2003) studied the impact of land-use change on soil properties (pH, organic matter (OM), phosphorus (P), and potassium (K)) between 1985 and 1997 in a hill region of subtropical China [15]. Guo et al. (2018) studied the relationship between vegetation restoration and soil quality (pH, nitrogen (N), P, K, etc.) in the loess hilly region of China [16], which has a semiarid climate. Wu et al. (2019) quantified 10 soil physical–chemical indicators in the Yellow River delta in 2014 [17]. Shuai Chen et al. (2020) studied the spatial–temporal change in soil chemical properties (pH, OM, P, K, and N) in Jianli county, which is located in the Han River plain [11]. The climate and agricultural activity in mountainous areas are different from those of plains. To fill the knowledge gap, it is essential to identify the spatial–temporal pattern of soil chemical properties in subtropical mountainous areas and identify its impact on China.

This study’s objectives are to (1) explore the temporal and spatial changes in soil chemical properties in mountainous areas with a subtropical continental monsoon climate, and to (2) explore the relationship among soil chemical properties, topographic factors, soil texture, and cropping systems. This research could be a reference for local agricultural managers and soil studies in mountainous areas.

## 2. Materials and Methods

### 2.1. Study Area

**Figure 1:**
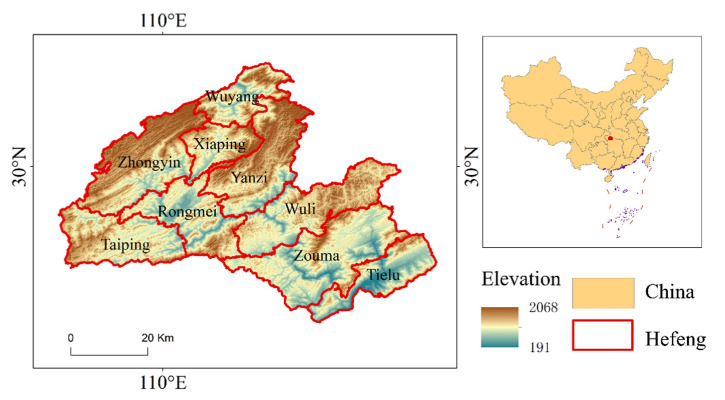
Elevation of Hefeng County, China.

Hefeng County (109°45′ E–110°38′ E, 29°38′ N–30°14′ N), located on the southwestern border of Hubei, at the edge of the Yungui Plateau mountain range, represents the northern section of the Shi Men branch of the Wuli Mountains (WL) (Figure 1). This area is composed of complex terrain; the elevation ranges from 191 to 2068 m with an average of 1147 m, and the average slope is 24.1°. In total, 80% of the study area is higher than 800 m. Hefeng also has a complicated climate. The average annual temperature is 15.5, 12.2, and 9.8 °C in the low-altitude mountains (below 800 m), middle-altitude mountains (800–1200 m), and high-altitude mountains (above 1200 m), respectively. The frost-free period is 216–266 days per year, which is favorable for the growth of crops. The total area of cropland in Hefeng is 20.52 × 10^3^ km^2^, of which 13.44 × 10^3^ km^2^ is a tea plantation. Fields are cultivated with annual crops, including rice, maize, potatoes, and sweet potatoes. Maize is the most cultivated, followed by potato and rice. The paddy fields are mainly composed of retention-type rice soils, with a total area of 2470 hectares, accounting for 12% of the county’s arable land and 90% of the paddy fields; the drylands are dominated by yellow-brown soils, accounting for 61.2% of the dryland area [18].

**Figure 2:**
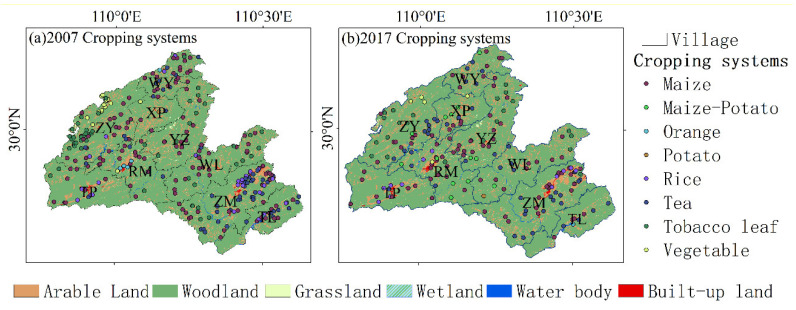
Cropping system distribution maps of the study area in (**a**) 2007 and (**b**) 2017.

The study area has a temperate continental monsoon climate. The annual precipitation (1701 mm) is distributed mainly from June to September [19]. According to the Second State Soil Survey of China (SSSSC, 1982), there are 10 main soil types in Hefeng County (Figure 2): rice soil, tidal soil, limestone soil, purple soil, red soil, yellow soil, yellow-brown soil, meadow soil, swamp soil, and brown soil. The most widely distributed soil in this area is the mountainous yellow-brown soil, which is weakly acidic but high in soil fertility.

Based on a comparison with the SSSSC report of 1982, the chemical properties of cropland soils in Hefeng County changed greatly by 2007. The measurement techniques used in 2007 were the same for the SSSSC report. Soils were more acidic in 2007 than in 1982. The soil with pH less than 5.50 accounted for 6.0% and 66.3% of all cultivated land in 1982 and 2007, respectively. The average value of OM, AK, and AP decreased from 32.2 g/kg, 148.3 mg/kg, and 1.7 mg/kg in 1982 to 31.2 g/kg, 116 mg/kg, and 45.9 mg/kg in 2007, respectively. The average value of soil AK and AP content decreased in the period of 1982–2007 [20].

### 2.2. Sampling Design and Laboratory Analyses

Three large-scale soil sampling activities have been carried out in Hefeng County, namely, the SSSSC, the soil for formulated fertilization test (2007), and the soil series survey (2017). Because these projects were carried with different standards, we only used the official report of the SSSSC in Hefeng and the soil chemical data with a location in 2007 and 2017. Moreover, the common indicators in the database of chemical properties of the soil sample sites for these two years were soil organic matter content, soil available phosphorus content, soil available potassium content, and soil pH. Therefore, we only studied the spatial distribution of these soil chemical properties in 2007 and 2017 and took the SSSSC report in Hefeng as a reference.

Soil sample locations were selected based on spatial homogeneity, soil type, and crop type. Sampling locations were determined using a Garmin GPS Etrex10 receiver. Samples were taken after harvest in November 2007 (290 samples) and November 2017 (200 samples) when no fertilizer or pesticides were applied in the cropland, which would ensure that environmental noise and systematic errors in the soil samples were eliminated as much as possible (Figure 2). Interviews with landowners were conducted at each sample site, and information was collected on their agriculture management practices such as crop rotation, yield, and irrigation. 

At each location, a soil drill was used to collect five topsoil samples (0–20 cm below the surface) according to the “S” method. Five topsoil samples were mixed to obtain composite soil samples. Each soil sample (>1 kg) was placed in an aluminum box, which was then sealed. Soil organic matter (OM) was tested by dichromate wet combustion. Available phosphorus (AP) was determined by extracting samples with 0.5 mol L^−1^ sodium bicarbonate (NaHCO_3_) and subsequent colorimetric analysis [21]. Available potassium (AK) was extracted with 1 mol/L NH_4_Ac, and then measured by an atomic absorption spectrometer [22]. Soil pH was determined by a pH meter (Sartorius Basic pH meter PB-10, Gottingen, Germany) with a soil/water ratio of 1:2.5 [23]. These same methods were used for both 2007 and 2017.

### 2.3. Data Analysis

#### 2.3.1. Descriptive Statistics and Difference Tests

The soil sample collection and agrochemical analysis are affected by uncertain factors, resulting in gross errors in soil’s measurement. Gross errors are often manifested as high or low values that clearly deviate from the data, thereby affecting the overall distribution characteristics and statistical analysis of the sample. We used the 3σ rule for outlier detection [24]:

(1) $$P\left\{\;\left|X_{i,j}-E\left(X_{j}\right)\;\right|>3\sigma \;\right\}=0.3\%$$

where $X_{i,j}$ is the value of sample i and soil property j; $E\left(X_{j}\right)\;$ is the average value of the whole sample of the soil property j; and σ is the standard deviation of the whole sample of the soil property j. If $X_{i,j}$ is lower than $E\left(X_{j}\right)$ − 3σ or higher than $E\left(X_{j}\right)$ + 3σ, then it is deleted when analyzing the data.

The sample numbers after outlier detection were 271, 263, 265, and 271 in 2007, and 192, 191, 183, and 184 in 2017, for OM, AP, AK, and pH, respectively. We used SPSS 25.0 for soil sample statistics, including mean, maximum, minimum, standard deviation, coefficient of variation, bias, kurtosis, and significance tests (Kolmogorov–Smirnov test, *p* < 0.05). Before spatial interpolation, we needed to assure that the soil variables were continuous and normally distributed. If the variables are not normally distributed, the semivariance function will be distorted, which reduces the precision of the interpolation results. Before spatial interpolation, we used a histogram, a normal Q–Q plot, and Kolmogorov–Smirnov tests to check whether the data were normally distributed. When the test results did not conform to the normal distribution, we performed either a logarithmic transformation, square root transformation, or Box–Cox transformation on the data. When the data were close to the normal distribution, we performed spatial interpolation.

Soil texture is influenced by topography, soil texture, and human activities. Five topographic factors were selected in this study, namely, elevation, slope, topographic relief, profile curvature, and planform curvature, to analyze the correlation between topography and soil texture by Pearson’s correlation [25] in SPSS 25. We used a 100 × 100 m moving window in the focal statistics toolkit in ArcGIS 10.7 to generate the raster for maximum and minimum elevation within a 100 × 100 m moving window. The topographic relief of cell i was the maximum elevation in cell i minus the minimum elevation value in cell i.

The profile curvature affects the acceleration and deceleration of flow and; therefore, influences erosion and deposition. The planform curvature influences convergence and divergence of flow. A negative value indicates that flow will be decelerated on the surface, while a positive profile indicates that the flow will be accelerated. A value of zero indicates that the surface is linear. The planform curvature is perpendicular to the direction of the maximum slope. The planform curvature relates to the convergence and divergence of flow across a surface. A positive value indicates that the surface is laterally convex at that cell. A negative plan indicates that the surface is laterally concave at that cell. A value of zero indicates that the surface is linear [26,27,28].

The topographic factors were extracted from the digital elevation model (Shuttle Radar Topography Mission, 30 m [29]) by the Spatial Analysis Extension in ArcGIS 10.7; four main soil textures of Hefeng County and eight cropping systems were used to analyze their relationship with soil chemical properties by one-way ANOVA using SPSS 25 [30,31]. The selected soil texture includes light loam, medium loam, sandy loam, and sandy soil. The cropping system is indicated in Figure 2.

#### 2.3.2. Geostatistical Analysis

(1) Semivariance

We used GS + 9.0 (Gamma Design Software, LLC, Plainwell, MI, USA) [32] to calculate each indicator’s semivariance and used ArcGIS 10.7 (Esri’s GIS mapping software, Esri, Redlands, CA, USA) to map the soil chemical properties. The semivariance function is used in geostatistics to quantitatively describe the spatial variation structure. The equation of the semivariance function is as follows:

(2) $$\gamma \left(h\right)=\frac{1}{2N\left(h\right)}\sum_{i=1}^{N\left(h\right)}[z\left(x_{i}\right)-z\left(x_{i}+h\right)]²$$

where γ(*h*) represents the semivariance with the lag distance (h), N(h) is the sample pair’s number within the lag distance h, z(x_i_) is the value of sample i, and z(x_i_ + h) is the value of the sample at distance h to sample i. We used the best-fit semivariogram models for each soil property, such as a stable model, circular model, Gaussian model, exponential model, or spherical model. Values were calculated for each possible pair of observation points, and the mean values of the semivariance were displayed for chosen distance intervals to produce the experimental semivariogram [33]. The sill (C0 + C) is the constant value when the semivariance function γ(h) increases with the lag distance h and reaches a relatively stable constant from the nonzero value. The nugget is the value when the lag distance h = 0, γ (0) = C0. The largest variation in the system and the lag distance α when the semivariance function γ(h) reaches the base station value are called the base station value and range, respectively. C0 represents the spatial heterogeneity of the random part, and C represents the spatial heterogeneity of system variation; the nugget-to-sill ratio (C0/C0 + C) was used to define different spatial class dependencies for the soil properties. C0/C0 + C was used to define different classes of spatial dependence for the soil properties. C0/C0 + C < 0.25, 25–0.75, and >0.75 were classified as having strong, moderate, and weak spatial dependence, respectively [11]. The stronger the spatial dependence in one soil property, the lower the C0/C0 + C value and the less random factor interruption (such as changes in cropping management and fertilization) experienced by the soil property [11].

(2) Spatial Autocorrelation

Analysis is a method of examining the autocorrelation between a regional variable and its neighboring variables. It determines whether there is spatial autocorrelation by detecting the dependence of a position variation on its neighboring positions [34]. We used global Moran’s I index and the Z scores to detect the spatial correlation between the variables. The equation of Moran’s I index is as follows:

(3) $$I=\overset{n}{\underset{i-1}{\sum }}\overset{n}{\underset{i=1}{\sum }}W_{i,j}(X_{i}-\;\bar{X})\left(X_{j}-\;\bar{X}\right)/S^{2}\overset{n}{\underset{i-1}{\sum }}\overset{n}{\underset{i=1}{\sum }}W_{i,j}$$

where I is global Moran’s I index, n is the number of samples, X_i_ and X_j_ are the samples’ property value at locations i and j, $\bar{X}$ is the mean of samples, and W_i,j_ is the weight based on the distance between samples i and j. The value of Moran’s I varies from −1 (negative autocorrelated) to 1 (positive autocorrelated). When the value of I equals 0, there is no autocorrelation between sample i and sample j.

Z scores (standard scores) can test whether the spatial autocorrelation is significant [35]. The formula is as follows:

(4) $$Z=\frac{x-\mu \;}{\sigma }∼N\left(0,1\right)$$

where x is the value of sample, μ is the mean value of the population, and σ is the standard deviation of the population. The Z score can determine the degree of spatial autocorrelation, and the larger the absolute value, the greater the spatial correlation; on the contrary, the smaller the absolute value, the smaller the spatial correlation. When the Z score is close to 0, it indicates that the space of the regional variable is randomly distributed.

(3) Fractal Dimensions

We used fractal dimensions (FD) to describe the spatial variability of soils. Fractal dimension is a measure of self-similar complexity [36]. The higher the FD value, the more complicated the landscape. It is used to describe the spatial correlation characteristics of soil chemical properties. Burrough introduced this fractal theory into soil science in 1983 and identified that different soil properties have different fractal characteristics [37]. The soil fractal dimension (D) is calculated as follows:

(5) $$\mathrm{FD}=2-H;H=\frac{1}{2}\log\gamma \left(h\right)$$

where FD means fractal dimension, FD ∈ [1,2], and H is the slope of the linear regression of log γ(h) ∝ log h in the scale h,H ∈ (0,1). FD = 2 indicates that there is no spatial auto-correlation in the distribution of the soil property, while FD = 1 indicates that there is a strong spatial auto-correlation in the distribution of the soil property [38].

In this study, we combined semivariance, spatial autocorrelation, and the fractal dimension to find the spatial variability of soil properties from different perspectives. There are differences in the calculation methods of semicovariance function and spatial autocorrelation. By calculating variance, the semivariance function quantifies the degree of spatial autocorrelation and the scope of the spatial variability scale of regional variables, and it provides the basis for kriging interpolation. The disadvantage of the semivariance function, however, is that it cannot provide statistical tests for significant and positive or negative spatial correlation [33]. This problem can be solved by the spatial autocorrelation index, which calculates covariance and makes up for the lack of significance tests in the semivariance model. Since the fractal dimension is a dimensionless composite indicator [38], it can be used to reveal spatial heterogeneity and provide empirical evidence for spatial autocorrelation and variance functions. Each of these three analytical tools has its own advantages and disadvantages, and they are used in combination to complement each other. The three tools are combined to comprehensively analyze the spatial structural characteristics and variation patterns of soil chemical properties.

(4) Spatial Interpolation

Spatial interpolation is the main way to identify the changes in the spatial distribution pattern of soil nutrient content. This research uses kriging interpolation [39,40], the most commonly used in soil science [41], to explore the spatial distribution of soil chemical properties in Hefeng. The method is based on semivariogram theory and structural analysis to predict unsampled points. We used GS+9.0 to fit the optimal half-variance theoretical model combined with ordinary kriging interpolation in ArcGIS 10.7 for spatial interpolation. We verified the interpolation accuracy of this study, as shown in Table 1. The results indicate that the interpolation accuracy is high. The root mean square error (RMSE) of each property was higher than one in the study period, which means that the interpolated value is positively related to the real value. The standard error of mean (SEM) of every property is close to 0. The value of the root mean square error (RMSE) of each indicator is similar to the value of the mean error (MAE) [42,43], and the standard root mean square error (RMSS) of different soil properties is close to one. The values of RMSE, SEM, RMSS, and MAE all prove that the interpolation has reasonable accuracy (Table 1). We classified each soil property in the maps according to the standard of the SSSSC [44,45] (Table 2), which is the official standard of soil property classification in China. It has been often referred to by other researchers [46,47,48,49,50] and is the geochemical survey standard used in China [51].

## 3. Results

### 3.1. Descriptive Statistics and Difference Tests

#### 3.1.1. Descriptive Statistics

In the K–S (Kolmogorov–Smirnov) test, only when the *p*-value is higher than 0.05 are the data normally distributed. We transformed all original soil data with a natural logarithm transformation, because in the K–S test, the *p*-value of all of the chemical soil properties was lower than 0.01.

The mean value of soil OM, AP and pH in Hefeng increased from 33.64 g/kg, 39.56 mg/kg, and 5.36 in 2007 to 35.49 g/kg, 58.51 mg/kg, and 5.99 in 2017, respectively (Table 3). The mean value of soil AK decreased from 147.82 mg/kg in 2007 to 118.02 mg/kg in 2017. Moreover, the maximum value of all indexes except pH decreased. The minimum value of all indexes increased except AK. The standard deviation (SD) and coefficient of variation (CV) value of all of the soil properties declined in the period of 2007–2017, which means that the spatial variation of each soil property decreased. The CV values of all indexes in both years are higher than 10%, which means that each index is moderately variable [52]. The AP in 2007 had the highest variability among all of the indicators, with a CV value of 76.46%.

In 2007, soil pH is poorly correlated with other soil chemical properties. Nevertheless, soil OM is significantly positively correlated with soil AP (r = 0.192, *p* < 0.01) and soil AK (r = 0.245, *p* < 0.01). Soil AP is significantly positively correlated with soil AK (r = 0.241, *p* < 0.01). In 2017, AP and AK no longer correlate well with OM, and AP is poorly correlated with AK. The correlations between other indices are significant but generally weak (*p* < 0.05) (Table 4).

#### 3.1.2. Pearson’s Correlation between Topographic Factors and Soil Chemical Properties in Hefeng

According to Pearson’s correlation analyses, in 2007 and 2017, OM (r = −0.197, *p* < 0.01) and AP (r = −0.043, *p* < 0.01) had a negative relationship with elevation. However, the other topographic factors had limited effects on OM and, with both negatively related to the slope (r = −0.159, *p* < 0.05) and planform curvature of land (r = −0.219, *p* < 0.05) in 2007 and AP negatively related to the slope in 2017 (r = −0.154, *p* < 0.01). AK positively was related to elevation in 2007 (r = 0.305, *p* < 0.01) and 2017 (r = 0.408, *p* < 0.01) and negatively related to topographic relief (r = −0.173, *p* < 0.0) and profile curvature (r = −0.175, *p* < 0.05) in 2017. The pH was negatively related to planform curvature of land in 2007 (r = −0.227, *p* < 0.01) and 2017 (r = −0.128, *p* < 0.01). In conclusion, the soil at a higher altitude was lower in OM and AP but high in AK. The soil located on steeper land had a lower AP and lower AK, while soil on land with high topographic relief, profile curvature, and planform curvature had less AP, AK, and pH (Table 5).

#### 3.1.3. Soil Chemical Properties in Different Soil Texture

The soil chemical properties in the various soil textures were different (Table 6). Soil OM and AK in 2007 are significantly (*p* < 0.05) correlated with soil texture (*p* < 0.05). On the other hand, soil AP and soil pH in 2007 and all soil chemical properties in 2017 are poorly correlated with soil texture. The OM content was highest in medium loam and lowest in sandy soil. The soil AP contents were highest in medium loam and lowest in sandy soil. The correlation between different soil textures and soil fertility content decreased in the period of 2007–2017 (Table 6).

#### 3.1.4. Soil Chemical Properties in the Different Cropping Systems

**Figure 3:**
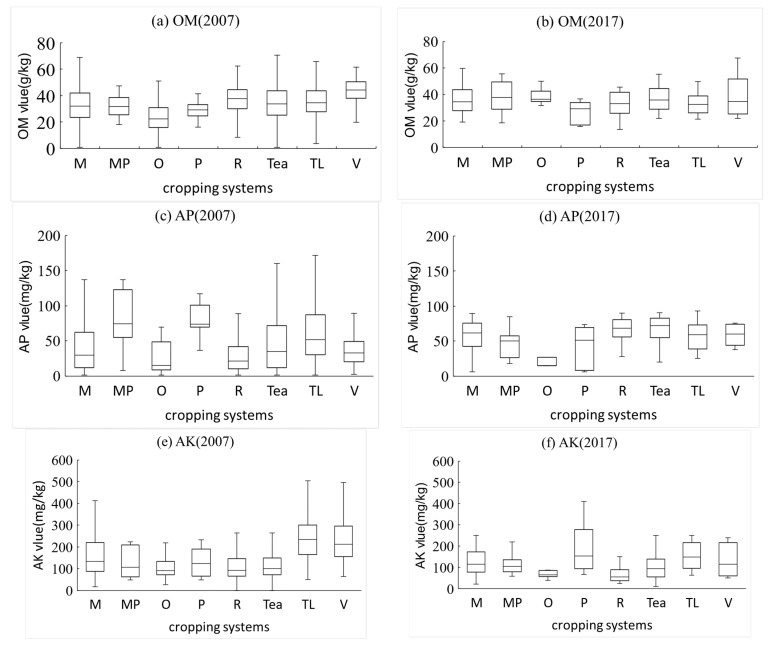

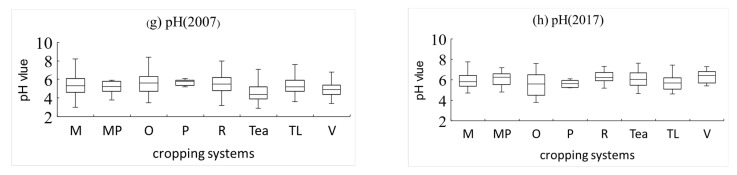
Box plots of contents of soil properties grouped according to cropping systems. (M—maize; MP—maize–potato; O—orange; P—potato; TL—tobacco leaf; V—vegetable). The box plots depict the minimum, maximum, median, and upper and lower quartiles of the data for each cropping system. (**a**) organic matter value2007(OM2007), (**b**) organic matter value2017(OM2017), (**c**) available phosphorus2007(AP2007), (**d**) available phosphorus2017(AP2017), (**e**) available potassium2007(AK2007), (**f**) available potassium2017(AK2017), (**g**) pH(2007), (**h**) pH(2017).

With Figure 3, we were able infer that farmers plant the crop according to the soil chemical properties, and that farming activities also influence the variation of soil properties. The changes in soil chemical properties vary from crop to crop. The cropping systems were well correlated with soil chemical properties in 2007 (*p* < 0.01). On the other hand, we only observed a significant correlation between AK and cropping systems in 2017 (*p* < 0.05). Based on cropping system types, the highest mean OM values were recorded for the soil where vegetables (43.79 g/kg) and rice (37.38 g/kg) were planted in 2007 and the soil where vegetables (38.64 g/kg) and maize–potatoes (38.19 g/kg) were planted in 2017. The lowest mean OM value was measured in the soil where oranges (26.15 g/kg in 2007; 31.60 g/kg in 2017) were planted. The highest mean AP values were recorded for the soil where maize–potatoes (77.86 g/kg) and tobacco leaves (62.45 g/kg) were planted in 2007 and the soil where rice (67.47 mg/kg) and potatoes (66.00 mg/kg) were planted in 2017. The lowest mean AP values were measured in the soil where oranges (31.62 mg/kg) were planted in 2007 and maize–potatoes (46.85 mg/kg) were planted in 2017. The highest mean AK values were recorded for the soil where tobacco leaves (250.02 mg/kg in 2007; 149.85 mg/kg in 2017) and vegetables (239.55 mg/kg in 2007; 132 mg/kg in 2017) were planted. The lowest mean AK value was measured in the soil where oranges (111.82 mg/kg in 2007; 62.00 mg/kg in 2017) were planted. The highest mean pH value was recorded for the soil where rice (5.45) and potatoes (5.41) were planted in 2007 and the soil where rice (6.25) and vegetables (6.33) were planted in 2017. The lowest mean pH values were measured in the soil where tea (4.65) and potatoes (5.44) were planted in 2007 and where oranges (5.73) were planted in 2017 (Table 7).

### 3.2. Geostatistical Analysis

#### 3.2.1. Semivariogram and Spatial Autocorrelation

**Figure 4:**
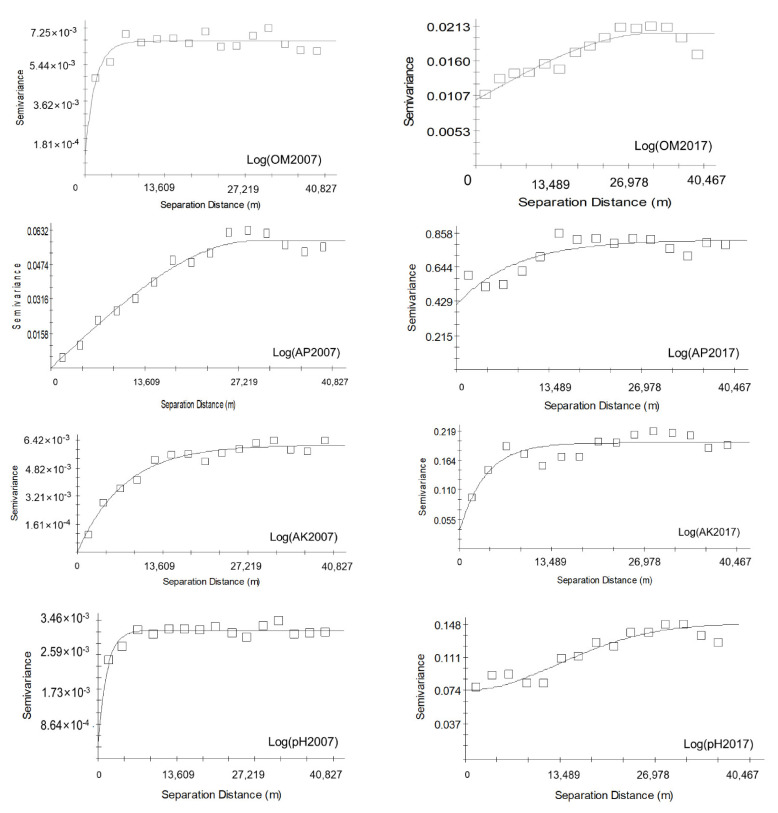
Experimental semivariograms for log (OM), log (AP), log (AK), and log (pH) in 2007 and 2017, Hefeng.

The most appropriate model was selected based on the maximum coefficient of determination (R2). R2 in this study ranges from 0.679 to 0.875, and the whole residual sum of squares (RSS) is lower than 0.001, which indicates that the semivariance models of each soil property are fitted (Table 8). With the exclusions of log (OM) in 2007, which was best fitted with the spherical model, and log (pH) in 2007 and 2017, which was best fitted with the Gaussian model, the soil chemical properties were best fitted with the exponential model in 2007 and 2017. The stronger the spatial dependence in one soil property, the lower the C0/C0+C value and the less random factor interruption (such as changes in cropping management and fertilization) experienced by the soil property. The spatial dependencies for all soil chemical properties were at the middle level, except AP (2007), pH (2007), and AK (2017). The value of C0/C0+C of these soil properties ranged from 0.350 to 0.500, which means that they were affected by both stable factors, such as topography and soil parent materials, and random factors, such as farming activities and climate. The nugget/sill values of AP (2007), pH (2007), and AK (2017) were lower than 0.17, which means that these factors were high in spatial dependency and were most likely affected by the natural environment. The semivariogram range value is the maximum distance within which autocorrelation or spatial dependence exists [53]. The ranges of soil properties in Hefeng varied from 15,630 (AK) to 41,673.142 m (OM) in 2007, and from 11,460 (AK) to 34,069.439 m (AP) in 2017. The spatial dependence of each soil property index showed a decrease during the period of 2007–2017. The Moran’s I index values ranged from 0.142 (AP) to 0.401 (pH) in 2007 and from 0.203 (AP) to 0.316 (AK) in 2017. The FD value was higher than 1.83 in all properties. The Moran’s I index values and FD value of OM and AP increased in the period of 2007–2017, while those of AK and pH decreased, which indicates that the spatial autocorrelation of OM and AK increased while that of AK and pH decreased. The Z value of all of the properties was higher than 1, which means that the soil chemical properties in Hefeng were spatially autocorrelated. In conclusion, the results of semivariograms, Moran’s I index, and FD are consistent (Table 8, Figure 4).

#### 3.2.2. Spatial Distribution of Soil Properties

In this study, based on the classification of soil chemical properties in the SSSSC (Table 3), we mapped the spatial variability of soil properties (OM, AP, AK, and pH) using the original kriging interpolation in 2007 and 2017. The spatial heterogeneity of soil properties in 2007 was more evident than that in 2017, and the spatial distribution of each soil property was different between 2007 and 2017.

(1) Spatial Distribution of Soil Organic Matter in 2007 and 2017

In 2007, approximately 0.30%, 0.51%, 5.89%, and 23.63% of the land were at the lowest, low, lower-medium or upper-medium level of soil AP, respectively. Moreover, 42.63% and 27.04% of the land were at the high and highest soil AP levels, respectively. In 2017, the proportion of land at the high level of soil AP increased by 26.15% of the total area in Hefeng, and the land area at the other level decreased. The CV for soil AP in Hefeng County revealed high-to-moderate variability (34.83% in 2007, 25.44% in 2017; Table 3).

**Figure 5:**
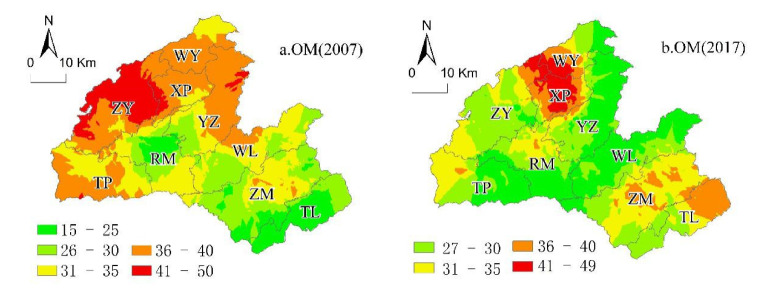
Ordinary kriging distribution maps for (**a**) 2007 OM and (**b**) 2017 OM.

The spatial distribution patterns of soil organic matter in 2007 and 2017 were inconsistent (Figure 5), indicating that soil organic matter content was more likely influenced by the natural environment and human activities, such as fertilizer application and agricultural management (Table 8). The soil with the highest organic matter in 2007 (Figure 5a) was located in Rongmei (RM), Tiehe (TH), Wu Yang (WY), Xiaping (XP), Yanzi (YZ), Taiping (TP), Wuli (WL), and Zouma (ZM).

In 2017 (Figure 5b), the soil with high organic matter content mainly was located in Taiping (TP), southern Wu Yang (WY), and northeastern Tielu (TL). The soil with lower and middle levels of OM was located in the northern part of Wu Yang (WY), the eastern part of Taiping (TP), the southern part of Rong Mei, the northeastern part of Yanzi (YZ), and the northeast part Wuli (WL).

(2) Spatial Distribution of AP in 2007 and 2017

In 2007, approximately 9.42% of the land was at the lowest level, and 25.14% of the land was at the medium level of soil AP. A total of 65.43% of the land was at a high soil AP. In 2017, the proportion of land at the highest level of soil AP increased by 47.55% of the total area in Hefeng, and the land area at the other level decreased. The CV for soil AP in Hefeng County had high-to-moderate variability (76.46% in 2007, 36.28% in 2017; Table 4).

**Figure 6:**
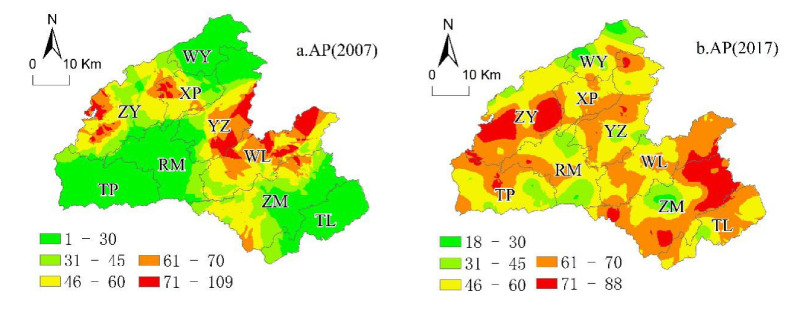
Ordinary kriging distribution maps for (**a**) 2007 AP and (**b**) 2017 AP.

In 2007 (Figure 6a), the soil AP in Hefeng County was lower in the southwest, southeast and north, including Taiping (TP), Tielu (TL), and northern Wu Yang (WY), followed by Rongmei (RM). The soil AP in Hefeng County was higher in Yanzi (YZ), Wuli (WL), Xiaping (XP), Zhongyin (ZY), and Zouma (ZM). In 2017 (Figure 6b), the soil AP was lower in northern Dongyang, southern Rongmei (RM), central Zouma (ZM), and eastern Taiping (TP). This part of the soil should be supplemented with phosphorus fertilizer; otherwise, the AP may become a limiting factor for crop growth. The soil with lower AP or higher AP in 2017 was associated with sandy loam or medium loam, respectively (Table 6).

(3) Spatial Distribution of Soil AK in 2007 and 2017

In 2007, approximately 21.69% and 47.24% of the land were at the low-to-medium level of soil AK. Moreover, 21.0% and 8.86% of the land were at the high and highest level of soil AK, respectively. In 2007, the proportion of land decreased by −0.68%, −12.71%, and −1.4% of the total area in Hefeng at the lower-middle level, upper-middle level, and high level, respectively. The proportion of land in low and lower levels increased by 3.03% of the total area in Hefeng, and the land area with soil AK higher than 200 mg/kg increased by 12.96% compared to 2007. The CV for soil AK in Hefeng County revealed a moderate variability (56.09% in 2007, 51.35% in 2017; Table 3).

**Figure 7:**
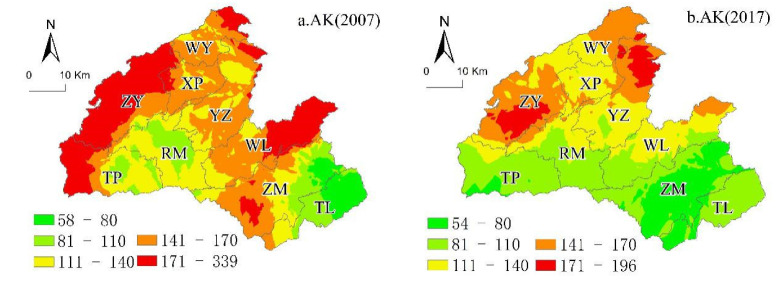
Ordinary kriging distribution maps for (**a**) 2007 AK and (**b**) 2017 AK.

From 2007 to 2017 (Figure 7), the land with soil AK greater than 50 mg/kg slightly decreased from 29.53 × 10^3^ to 26.61 × 10^3^ km^2^. The difference in the spatial variation of soil AK between the two periods was small. The spatial distribution of soil AK in 2007 (Figure 7a) in Hefeng County was generally from west to east, showing a high, medium, and low level of soil AK (Figure 7a). The soil with the highest values of AK was found in Zhongyin (ZY). The soil with higher AK levels was in Wuli (WL), and soil with the lowest levels of AK was in Zouma (ZM) and Tielu (TL). The average values of AK in soils in the remaining area were in the middle and upper levels. The spatial distribution of soil AK levels in Hefeng County in 2017 (Figure 7b) showed a continuous sheet-like distribution. The soil with the highest soil AK content was located in the north and central parts of Hefeng. The land with the lowest soil AK content was located in Zouma (ZM), Tielu (TL) Rongmei (RM), and Taiping (TP), while the soil with higher AK content was mainly located in eastern Wu Yang (WY), Yanzi (YZ), and Wuli (WL). Farmers in Zouma (ZM), Tielu (TL), and central Rongmei (RM) may increase the use of potassium fertilizer.

(4) Spatial Distribution of Soil pH in 2007 and 2017

In 2007, approximately 1.9%, 17.35%, 50.37%, and 27.95% of the samples were extremely acidic, very acidic, acidic, slight acidic, or neutrality, respectively, and 5.38% of the land was neutral. In 2007, the land proportion decreased by −1.03%, −16.17%, −37.58% at extremely acidic, very acidic, and acidic conditions, respectively, and the land proportion increased by 8.75% and 46.02% at slightly acidic conditions and neutrality, respectively. The CV for soil pH in Hefeng County had moderate variability (15.87% in 2007, 12.88 in 2017; Table 3).

**Figure 8:**
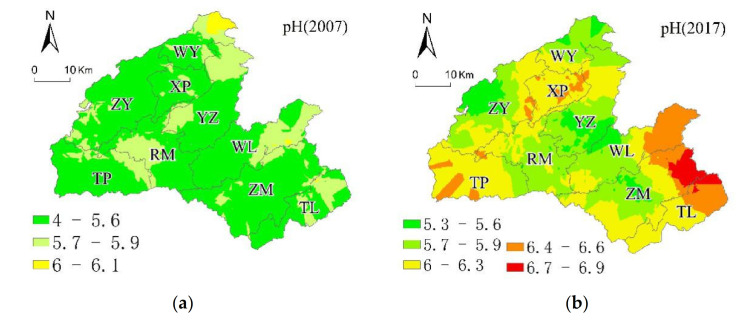
Ordinary kriging distribution maps for (**a**) 2007 pH and (**b**) 2017 pH.

The distribution of soil pH levels in Hefeng County was generally acidic, with pH levels at a neutral level in the southeast, north, and southwest in 2007 (Figure 8a). In 2017, 51.40% of the soil pH was at a neutral level, mainly in Zhongyin (ZY), Xiaping (XP), Rongmei (RM), and Zouma (ZM); 34.46% of the soil was weakly acidic. Soil below pH 5.5 was located in the northeastern part of Zhongyin (ZY), and the land of soil pH below 5.5 decreased from 50.37% of the total area in 2007 to 12.79% of the total area in 2017 (Figure 8b).

## 4. Discussion

Previous research shows that land-use change alters the ecosystem, resulting in variation in soil properties. Many factors influence the condition of soil properties, including human activities and natural elements. Natural conditions, such as soil parent material, topography, climate, and soil type, determine the main characteristics of soil properties. Human activities, such as arable land management, including fertilizer application, field cropping, and irrigation conditions, influence soil spatial–temporal variation [10,11,17,54,55]. Moreover, the methods of soil sample collection and processing also affect the precision of soil chemical property analysis by uncontrollable mistakes [5,56].

### 4.1. Possible Reasons for Soil Chemical Property Changes

Organic matter content is one of the core indicators that sustain soil life, and it is an essential determinant of soil fertility levels. According to the Hefeng soil chronicles [57], before 1970, farmers mixed river and lake mud or livestock manure with the soil to improve soil fertility. After reform and development, chemical fertilizers began to be used, and by 1990, soil fertility had declined due to the heavy use of chemical fertilizers. Our finding shows that the average soil OM in Hefeng was previously at a high level (Table 3 and Table 5), and it has risen during the period of 2007–2017 (33.64 g/kg in 2007 and 35.49 g/kg in 2017). Furthermore, the spatial difference (CV) of soil OM was high in both years, but it dropped from 34.83% in 2007 to 25.44% in 2017 with a higher area of continuous distribution of OM in the same grade in 2017 than in 2007.

Soil rich in OM (mean value of OM higher than 34.56 g/kg) may explain the type of terrain in Hefeng County, where elevation is high and evaporation is low. With high humidity in Hefeng, soil microbial activity is hindered. This result is similar to that of Yu Fang et al. (2019) [58]. In their study, the spatial heterogeneity of soil OM in western Hubei, where Hefeng is located, was high. There are multiple possible reasons for the changes in soil OM from 2007 to 2017. The first likely reason is the decrease in farmland-use intensity. According to the yearbook of Enshi [59,60], the multiple crop index (the acreage of sowing divided by the acreage of arable land) dropped from 2.83 in 2007 to 2.39 in 2017, which means that the frequency of agricultural activities has also dropped. Soil OM may be influenced by fallow frequency [61,62]. The second possible reason is that, in recent years, the Hefeng County agricultural department has implemented a soil organic matter improvement subsidy program to improve soil fertility, and it strongly supports organic fertilizers [63]. These actions may have changed the soil OM content. The third reason may be the variation in cropping systems. According to the yearbook of Enshi [59,60], the acreage of each crop has changed, in which the grain area and sowing area for vegetables have risen from 239.1 to 243.1 km^2^ and from 75.6 to 85.1 km^2^, respectively. The variation in sowing acreage of different crops was affected by the agricultural market and national policies [64,65]. The soil in crop systems, including maize, rice, tobacco leaves, and vegetables, has a relatively high OM content (Table 7, Figure 3), which is attributed to the vegetation residual and cropping management [66,67]. The frequency and intensity of activities such as fertilization, seeding, and loosening of farmland soil differ by crop, thereby causing the differences in soil OM in farmland. The increased soil OM may also be related to the increased acid rain in western Hubei, according to Wu et al.’s research (2016) [55].

Average soil AP value increased from 39.56 mg/kg in 2007 to 58.51 mg/kg in 2017. This trend corresponds with the soil AP in paddy fields in southern China [11]. A total of 86.72% of the land in Hefeng had AP content higher than 40 mg/kg in 2017. The Zouma (ZM) phosphate mine in Hefeng has a reserve of 1.178 billion tons, making it one of the four largest phosphate mines in China [68]. The wastewater, exhaust gas, and sludge from phosphate mines have a negative impact on the surrounding environment, especially in mining areas with steep slopes and topography, which are prone to landslides and severe soil erosion during mining, and this could consequently pollute the surrounding land [69]. According to the yearbook of Enshi [59,60], AP fertilizer application decreased from 6233 tons in 2007 to 5488 tons in 2017, but the pollution is still severe.

Soil in Hefeng was at the upper-middle level of AK with a mean value at 147.82 mg/kg in 2007, and it dropped to 118.02 mg/kg in 2017. We learned from a survey of farmers in Hefeng that although little potash is contained in the straw returned to the field, farmers have long neglected the use of potash, leading to a reduction in soil AK. The low application rate of K fertilizer is also reported in previous research [11,70]. According to these studies, intensive farming was also one of the main causes of soil potassium depletion [71].

Soil pH affects soil microbial activity, plant growth, and nutrient transfer [72]. However, in our study, soil pH in Hefeng is poorly correlated with AK, AP, and OM (Table 4). The influence of coal mines and the pollution of exhaust vapor from factories (such as sulfur dioxide from sulfur and phosphate fertilizer plants) cause low soil acidity in nearby land. For example, near the sulfur plant in Erdeng village, the soil acidity is lower than 4.5, and no plants grow there except for millennium dwarfs and tea trees [57]. Soil acidity affects the effectiveness of nitrogen, phosphorus, and potassium nutrients. The AP in the soil is highest when the pH value ranges from 6.0 to 7.5, and AK is highest when the pH value between 6 and 10. When the soil is too acidic, plant roots rot [72].

Soil acidification is the main problem in Hefeng County, which is different from soil pH in the grain production area of China’s subtropical plain, where soil is alkaline [11]. However, soil pH changes in the study area and the soil in central China’s plain area were similar. The pH value of these two regions increased during the period of 2007–2017 (Table 3) [11]. The average soil pH in Hefeng increased by 0.63 or 11.75% (from 5.36 to 5.99) (Table 3), and the soil changed from acidic to weakly acidic. The reason for this phenomenon is because the pH of rainfall in Hubei province has increased. According to Wang et al.’s (2016) research [73], acid rain (the pH value of the rain less than 5.6) frequency in the southwestern part of Hubei province dropped by more than 50% during the period of 2007–2014. The acreages of ecological tea plantations in Hefeng County are similar to those of arable land, and tea plantations require acidic soil. Our field survey [71] showed that in recent years, farmers have been neutralizing the land where soil acidification is severe. Local farmers use lime to moderate the soil’s acidic quality and ease the problem of soil acidification, thereby allowing the soil to become loose and breathable, and providing it with an increased ability to retain water, warmth, and fertilizer. 

We explored the interaction among soil chemical properties, topographic parameters, and soil texture in Hefeng County using SPSS 25. The finding shows (Table 4) that in 2007, soil pH did not exhibit a correlation with OM, AP, or AK. Soil OM was significantly positively correlated with AP (r = 0.192, *p* < 0.01) and AK (r = 0.245, *p* < 0.01). Soil AP was significantly positively correlated with AK (r = 0.241, *p* < 0.01). In 2017, AP and AK no longer correlated well with OM. AP was poorly correlated with AK. The correlations between other indices were significant but generally weak (*p* < 0.05, R2 < 0.6). The soil OM (r = −0.197, *p* < 0.01) and soil AP (r = −0.043, *p* < 0.01) decreased at higher altitudes but exhibited higher AK. The soil located in steeper land had lower AP and AK. The soil in areas with high topographic relief, profile curvature, and planform curvature had less AP and AK, and lower pH (Table 5). 

The OM and soil AK in 2007 were significantly correlated (*p* < 0.05) with soil texture (*p* < 0.05), while soil AP and soil pH in 2007 and all soil chemical properties in 2017 were poorly correlated with soil texture (Table 6). The OM content was highest in medium loam and was lowest in sandy soil. The soil AP content was highest in medium loam and lowest in sandy soil. The correlation between soil textures and soil chemical properties decreased from in the period of 2007–2017. The cropping systems played an important role in affecting soil chemical properties in 2007 (*p* < 0.01), while they only significantly affected AK in 2017 (*p* < 0.05).

### 4.2. Suggestions for Local Farming Management

Since soil chemical properties not only interact with each other, but are also influenced by soil texture, soil cultivation activities, cropping systems, and topographical factors, this research leads to the following suggestions:

#### 4.2.1. Moderate Use of Fertilizer

Fertilizing according to the soil condition—it is advisable to apply semi-rotten fertilizers on sandy soils, and quick-acting fertilizers should be applied several times in small amounts to prevent fertilizer shedding.

Maintaining and improving soil organic matter [62]—the application of organic fertilizers or the application of organic fertilizers with chemical fertilizers can maintain the soil acid–base balance and slow soil acidification, because organic fertilizers contain alkaline substances. However, as livestock manure may contain pollutants such as heavy metals and antibiotics [74,75], care should be taken to avoid environmental and health risks when choosing organic fertilizers and to avoid the addition of harmful substances into acidic soils.

Neutralizing acidified soil—the common method to improve acidic soil is to apply alkaline substances such as lime [76] or straw-return to the field [77,78], but the cost of this is high [79]. From the long-term perspective, large applications of lime can lead to soil slumping and nutrient imbalance, because lime only provides the nutrient calcium, and large amounts of calcium can decrease soil effectiveness of magnesium (Mg), potassium (K), and phosphorus (P) [80].

Despite the slow decrease in the amount of phosphorus fertilizer applied in recent years (from 13,000 in 2007 to 9900 t in 2017, according to the yearbook of Enshi [59,60]), it is still applied in generally large quantities. The excessive use of phosphorus fertilizer leads to increased costs for farmers and soil environmental pollution problems; thus, it is recommended to use phosphorus fertilizer sparingly to reduce the accumulation of soil AP. Moreover, it is also necessary to control soil contamination caused by factories [81].

At present, there is a problem of soil acidification in Hefeng County, and it should be noted that the tea industry in Hefeng County ranked number one among the tea industries in Hubei Province. The soil of tea plantations is acidic; thus, soil acidification in this study area should be considered with the local situation when improving the pH of the soil.

#### 4.2.2. Crop Selection According to Soil Suitability and Topographic Factors

While acidic (or alkaline) fertilizer can neutralize the properties of soil, it has a high cost [82]. In acidic soil, farmers can plant tea, oil tea, sweet potatoes, yams, buckwheat, and citrus fruits [83]. In land with different topographic relief properties, food crops can be planted on flat fields. In middle hills, farmers can plant tea, oil tea, and other specialty products. In general, famers can plant green manures and winter crops, increase potassium fertilizer, and coordinate the nutrient ratio in order to improve soil fertility.

#### 4.2.3. Construction of Water Conservancy Facilities

Improving water irrigation facilities can reduce the changes in drought and flooding, and also curb soil erosion on sloping land. The shallower the cultivation layer, the thinner the soil layer. For places that are less steep, terraces can be built.

Although the amount of applied potassium fertilizer is high (an increase of from 1753 tons in 2007 to 4505 tons in 2017), soil erosion in mountainous areas may lead to potassium loss [84] (Table 5). Soil potassium remains a limiting factor for crop growth in Hefeng.

Local agricultural departments should help famers to choose proper amounts of fertilizer and construct more facilities for farmland water conservation. The government should regulate mining facilities to prevent them from negatively affecting soil. 

## 5. Conclusions

This research investigated the spatial changes in soil OM, AP, AK, and pH content in Hefeng in 2007 and 2017 by using the geostatistical methods incorporated in Gs + 9.0 and ArcGIS 10.7.

The results show that the mean value of soil OM, AP, AK, and pH in Hefeng increased from 33.64 g/kg, 39.56 mg/kg, 147.82 mg/kg, and 5.36 in 2007 to 35.49 g/kg, 58.51 mg/kg, 118.02 mg/kg, and 5.99 in 2017, respectively. The spatial variation and dependency of each soil property were at the middle level, which means that they were affected by both stable factors, such as topography and soil parent materials, and random factors, such as farming activities and climate.

In conclusion, the findings of this study in combination with those of others suggest that the topography and soil texture, acid rain, and farmers’ fertilization practices in Hefeng are all related to the chemical properties of soil and its temporal and spatial variation.

Because of the different soil survey standards in Hefeng, only a few items of variable soil chemical data can be represented for a long-time scale. Our research is limited to analyzing the impact of soil properties from the climatic prospective due to lack of temperature and precipitation data at different elevations in Hefeng County. Moreover, we did not analyze the spatial variance of the correlation of topological factors, soil types, soil texture, and soil chemical properties. Further work could be conducted by quantifying the relationship between changes in soil chemical properties with climatic factors and social economic factors. However, the existing data are still essential for understanding soil chemical property changes in this subtropical mountainous region. With stricter procedures of soil sampling, testing, and additional regular soil surveys and soil monitoring, our future studies in Hefeng will be more in-depth, precise, and complete.

## Figures and Tables

**Table 1:** Accuracy of soil data interpolation in Hefeng County.

Soil Property	Year	RMSE	SEM	RMSS	MAE
OM	2007	10.216	0.0021	1.2248	9.545
	2017	8.629	0.0260	1.0046	9.295
AP	2007	35.687	−0.0206	0.9843	36.354
	2017	21.971	−0.0193	0.8969	28.803
AK	2007	68.447	−0.0216	0.9236	88.789
	2017	55.984	−0.0037	0.8401	66.949
pH	2007	0.903	0.0034	0.997	0.956
	2017	0.8034	0.0059	1.119	0.831

Note: OM—organic matter, AP—available phosphorus, AK—available potassium, RMSE—the root mean square error, SEM—standard error of mean, RMSS—the standard root mean square error, and MAE—the value of the mean error.

**Table 2:** Classification of soil chemical properties in the Second State Soil Survey of China.

Level of pH	pH	Level of AP, AK, and OM	AP	AK	OM
Extremely acidic	<4.5	Very low	<3	<30	<6
Very acidic	4.5~5.0	Low	3~5	30~50	6~10
Acidic	5.0~5.5	Lower-middle	5~10	50~100	10~20
Slightly acidic	5.5~6.0	Upper-middle	10~20	100~150	20~30
Neutrality	6.0~7.9	High	20~40	150~200	30~40
		Very high	>40	>200	>40

Note: OM—organic matter, AP—available phosphorus, AK—available potassium The unit of OM is g/kg; the unit of AP and AK is mg/kg.

**Table 3:** Descriptive statistics of the soil indicators.

	Year	Mean	Maximum	Min	SD	CV (%)	Skewness	Kurtosis	K–Sp
OM (g/kg)	2007	33.64	59.60	3.40	11.79	34.83	0.09	−0.23	<0.01
	2017	35.49	55.60	13.64	9.03	25.44	0.02	−0.28	<0.01
AP (mg/kg)	2007	39.56	158.10	0.10	37.60	76.46	−0.68	0.99	<0.01
	2017	58.51	89.40	6.10	21.23	36.28	0.02	−0.28	<0.01
AK (mg/kg)	2007	147.82	352.00	17.00	82.56	56.09	−0.40	−0.20	<0.01
	2017	118.02	250.00	10.00	62.97	51.35	−0.24	−0.08	<0.01
pH	2007	5.36	7.08	4.00	0.85	15.87	0.16	0.45	<0.01
	2017	5.99	7.75	4.63	0.77	12.88	0.04	−0.07	<0.01

Note: SD—standard deviation, CV—coefficient of variation; K–Sp—the *p*-value of K–S (Kolmogorov–Smirnov) test.

**Table 4:** The relationships among soil properties in Hefeng in 2007 and 2017.

	pH	OM	AP	AK	
pH_2007	1	−0.110	0.185 **	−0.062	pH_2017
OM_2007	−0.099	1	0.001	0.017	OM_2017
AP_2007	−0.017	0.192 **	1	0.035	AP_2017
AK_2007	0.087	0.245 **	0.241 **	1	AK_2017

Note: ** indicates significant correlation at the 0.01 level.

**Table 5:** Pearson’s correlation between topographic factors and soil chemical properties in Hefeng.

Topographic Factors	Year	OM	AP	AK	pH
Elevation	2007	−0.197 **	−0.043 *	0.305 **	−0.012
	2017	−0.334 **	−0.121 *	0.408 **	−0.089
Slope	2007	−0.037	−0.159 *	−0.015	−0.182
	2017	−0.021	−0.154 **	−0.175 **	−0.038
Topographic relief	2007	−0.071	0.114	0.062	−0.032
	2017	−0.022	−0.151 *	−0.173 **	−0.039
Profile curvature	2007	−0.070	−0.120	−0.005	−0.183
	2017	−0.022	−0.153 **	−0.175 **	−0.038
Planform curvature	2007	−0.028	−0.219 *	0.022	−0.227 **
	2017	−0.015	−0.114	−0.074	−0.128 *

Note: ** *p* < 0.01; * *p* < 0.05.

**Table 6:** Difference significance tests of soil properties between 2 crop years (2007an 2017) and 4 soil texture.

Soil Textures	2007				2017			
	OM (g/kg)	AP (mg/kg)	AK (mg/kg)	pH	OM (g/kg)	AP (mg/kg)	AK (mg/kg)	pH
Light loam	36.96	37.93	201.31	5.51	35.11	59.45	119.50	6.06
Sandy loam	35.77	36.42	181.21	5.12	41.94	59.60	134.92	5.55
Sandy soil	30.46	33.12	123.82	5.18	43.33	56.42	108.67	6.38
Medium loam	38.67	42.09	179.72	5.29	37.72	59.66	113.57	5.88
ANOVA (F values)	6.68 **	0.50	7.75 **	1.03	2.18	0.02	0.29	1.48
Years	0.003	9.585 *	61.097 **	146.878 **				

Note: ** and * indicate difference is significant at the 0.01 level and 0.05 level, respectively.

**Table 7:** Difference significance tests of soil properties between two crop years (2007 and 2017) and eight cropping systems.

Cropping Systems	2007				2017			
	OM (g/kg)	AP (mg/kg)	AK (mg/kg)	pH	OM (g/kg)	AP (mg/kg)	AK (mg/kg)	pH
Maize	34.46	44.12	178.46	5.3	35.90	57.44	125.41	5.93
Maize–Potato	32.48	77.86	127.08	5.18	38.19	46.85	112.62	6.02
Orange	26.15	31.62	111.82	5.39	31.60	61.65	62.00	5.73
Potato	28.68	78.84	142.5	5.41	36.55	66.00	104.00	5.44
Rice	37.38	32.14	125.12	5.45	32.87	67.47	67.15	6.25
Tea	35.19	48.97	127.04	4.65	36.51	64.44	106.26	6.11
Tobacco leaf	36.63	62.45	250.02	5.29	33.97	56.71	149.85	5.77
Vegetable	43.79	41.89	239.55	4.96	38.64	58.72	132.17	6.33
ANOVA (F values)	20.64 **	22.48 **	100.20 **	43.43 **	0.52	1.42	2.80 *	1.08

Note: ** and * indicate significant differences at the 0.01 and 0.05 level, respectively.

**Table 8:** Parameters of semivariograms, Moran’s I index, and fractal dimensions (FDs) of soil properties in 2007 and 2017, Hefeng.

Year	Soil Properties	Model	Nugget (C0)	Sill (C0 + C)	Nugget/Sill	Range (m)	R2	RSS	Moran’s I	Z	FD
2007	Log (OM)	S	0.010	0.020	0.498	41,673.142	0.860	2.23 × 10^−5^	0.235 ***	8.17	1.94
2017		E	0.004	0.008	0.500	22,320.000	0.833	1.15 × 10^−3^	0.243 ***	2.35	1.98
2007	Log (AP)	E	0.000	0.058	0.002	32,200.000	0.776	1.37 × 10^−4^	0.142 ***	4.92	1.834
2017		E	0.407	0.815	0.499	25,800.000	0.679	5.84 × 10^−2^	0.203 *	161	1.91
2007	Log (AK)	E	0.021	0.059	0.350	15,630.000	0.862	1.44 × 10^−6^	0.318 **	1.92	1.98
2017		E	0.032	0.197	0.165	11,460.000	0.718	4.19 × 10^−3^	0.316 ***	4.81	1.95
2007	Log (pH)	G	0.0002	0.001	0.143	34,467.811	0820	6.19 × 10^−7^	0.401 **	2.12	1.99
2017		G	0.0739	0.149	0.497	34,069.439	0.875	1.24 × 10^−3^	0.274 ***	3.15	1.99

Note: E—exponential; S—spherical; G—Gaussian. RSS—residual sum of squares, FD—fractal dimension value ***, **, and * indicate significant differences at the 0.01, 0.05, and 0.1 level, respectively.

## Data Availability

Not applicable.
